# Diverse vaginal microbiota in healthy Japanese women: a combined relative and quantitative analyses

**DOI:** 10.3389/fcimb.2024.1487990

**Published:** 2025-02-04

**Authors:** Masahiro Ito, Misaki Kataoka, Yoichi Sato, Hideki Nachi, Koji Nomoto, Nobuhiko Okada

**Affiliations:** ^1^ Laboratory of Microbiology, School of Pharmacy, Kitasato University, Tokyo, Japan; ^2^ Daikanyama Women’s Clinic, Tokyo, Japan; ^3^ HMS Women’s Health Research and Development Center, Hanamisui Co., Ltd., Tokyo, Japan; ^4^ Department of Molecular Microbiology, Tokyo University of Agriculture, Tokyo, Japan

**Keywords:** vaginal microbiota, viable bacterial counts, vaginal pH, relative bacterial abundance, lactobacillus, BMI, age, Japanese women

## Abstract

**Introduction:**

This cross-sectional study aimed to characterize the viable vaginal microbiota and identify host factors influencing this microbiota by employing a combination of relative and quantitative analyses.

**Methods:**

Twenty-four vaginal fluid samples were collected from healthy adult Japanese women for analysis. Vaginal fluid pH was measured using a portable pH meter. DNA was extracted from the vaginal fluid, and the 16S ribosomal RNA gene sequences in the V3-V4 regions were analyzed to identify bacterial species. Additionally, the vaginal fluid was cultured on four types of selective agar plates. The predominant species in the growing colonies were identified using colony polymerase chain reaction, and the colonies were counted.

**Results:**

The vaginal microbiota was classified into four categories based on the characterization of the dominant bacterial population: *Lactobacillus crispatus*, *Lactobacillus iners*, *Lactobacillus gasseri*, and a diversity group. The predominant bacterial species were consistent across methods; however, the levels of the viable population varied significantly. Body mass index had a significant influence on the total number of viable bacteria and vaginal pH, while age only affected vaginal pH.

**Conclusions:**

Our findings indicate that the vaginal microbiome of healthy Japanese women is not only highly diverse but also affected by host factors such as BMI and age.

## 1 Introduction

After puberty, healthy premenopausal women experience an increase in blood estrogen levels, which is associated with vaginal colonization by one or more *Lactobacillus* species, including *L. crispatus*, *L. iners*, *L. gasseri*, and *L. jensenii* (Ravel et al., 2011). Based on gene sequencing analysis, the vaginal community state type (CST) is classified into five groups according to its composition: CST I is dominated by *L. crispatus*, while groups II, III, and V are dominated by *L. gasseri*, *L. iners*, and *L. jensenii*, respectively (Ravel et al., 2011). CST IV is dominated by anaerobic bacteria instead of *Lactobacillus* (Ravel et al., 2011). Lactobacilli produce lactic acid as a metabolite (O’Hanlon et al., 2019), which lowers the vaginal pH and balances the vaginal microbiota that is rich in *Lactobacillus*, resulting in a high rate of spontaneous births (Kindinger et al., 2017; Ikumi and Matjila, 2022) and *in vitro* post-fertilization births (Moore et al., 2000; Kadogami et al., 2020, 2023). Hence, vaginal dysbiosis, characterized by a decreased presence of lactobacilli, is significantly associated with an increased risk of bacterial vaginosis (BV) (Vallor et al., 2001) and/or aerobic vaginitis (AV) (Jahic, 2022). This condition also increases susceptibility to infections such as human immunodeficiency virus (HIV) and human papillomavirus (HPV), as well as other sexually transmitted infections (Martin et al., 1999). Notably, *L. crispatus*, *L. gasseri*, and *L. jensenii* are not associated with diseases such as BV (Chee et al., 2020). *L. crispatus* is the most prevalent type of vaginal microbiota in Japanese women (Kurakawa et al., 2015). Additionally, *L. crispatus* is less likely to shift from its dominant composition compared to *L. iners*, which is another common type of vaginal microbiota (Gajer et al., 2012). Furthermore, women with *L. crispatus* dominance have been reported to have lower incidences of preterm birth and cervical dysplasia (Petricevic et al., 2014; Kindinger et al., 2017; Norenhag et al., 2020). These findings suggest that *L. crispatus* plays an important role in maintaining vaginal homeostasis.

The vaginal microbiota, consisting primarily of obligate anaerobes rather than lactobacilli, is characterized by a high species diversity (Ravel et al., 2011) and a significant presence of BV-associated bacteria, including *Gardonerella vaginalis* and *Prevotella bivia* (Gajer et al., 2012). BV could be asymptomatic; however, it is associated with serious complications in obstetrics and gynecology, such as preterm labor (Hillier et al., 1995; Petricevic et al., 2014) and sexually transmitted diseases (Wiesenfeld et al., 2003), including acquired immunodeficiency syndrome (Taha et al., 1998). Thus, the composition of the vaginal microbiota is closely related to host health and disease.

The vaginal microbiota has been extensively studied, mostly using 16S ribosomal RNA gene (rDNA)-targeted microbiota analyses. Although these methods effectively detect disease-associated microbiome variation in both vaginal and gut microbiota, previous research on the interaction between microbiota and host health has largely overlooked the possibility that changes in overall microbiota abundance could serve as a crucial indicator of a disease-associated ecosystem configuration (Harmsen et al., 2012; Satinsky et al., 2013). Therefore, it is essential to conduct microbiome studies based on viable bacterial counts rather than relying solely on compositional ratios to better understand host-microbe interactions (Props et al., 2016; Stämmler et al., 2016; Vandeputte et al., 2017). In this study, we aimed to analyze the vaginal microbiota via amplicon sequencing using next-generation sequencing to assess the relative compositional balance, along with employing a culture method using appropriate selective media to quantify bacteria in vaginal fluid. Additionally, we analyzed factors that may influence viable vaginal microbiota, such as vaginal pH, body mass index (BMI), age, the use of combined oral contraceptives (COC), and hormonal cycle.

## 2 Materials and methods

### 2.1 Participant selection criteria

The study was approved by the Research Ethics Committee of Kitasato Institute Hospital (Study No. 22024) and was performed per the principles of the Declaration of Helsinki. All the participants provided written informed consent. The inclusion criteria for this study were as follows: (1) women who were diagnosed as free of reproductive organ disease; (2) women who were not taking antibiotics or had not taken antibiotics in the past month; and (3) premenopausal women who were at least 18 years old or more. Vaginal fluid was collected from 30 participants between November 30, 2022, and March 22, 2023, at Daikanyama Women’s Clinic in Tokyo, Japan.

### 2.2 Participant details

Participant background information (age, height, weight, body temperature, place of birth/nationality, current medical history, medical history, medications/supplements/pills taken, allergies, history and type of pregnancy and delivery, pregnancy status, history of premature birth or miscarriage, menstrual cycle, duration of menstruation, and date of last menstruation) was collected using a questionnaire.

### 2.3 Collection of vaginal swabs and analysis of vaginal microbiota

Vaginal fluid was collected using two swabs from 30 participants, using the Copan eSwab (regular FLOQSwab; Copan Japan, Kobe, Japan), without considering the specific timing of their menstrual cycle phases. After collecting the vaginal swab, an eSwab was promptly pressed onto a sampling sheet. The pH value was measured using a pH meter (LAQUAtwin-pH-33B; Horiba, Kyoto, Japan). The sampling sheet was carefully placed on the pH sensor, which has a range of pH 0 to pH 14 and an accuracy of ± pH 0.01, according to the manufacturer’s instructions. Another was immersed in a tube containing 2 mL of the sample preservation solution and refrigerated. Vaginal swabs were shipped to the Laboratory of Microbiology, School of Pharmacy, Kitasato University, under refrigeration within 2 days. The sample preservation solution was divided into two parts. The vaginal microbiota was analyzed using (1) amplicon sequencing with a next-generation sequencer and (2) culture methods and colony polymerase chain reaction (PCR). DNA was extracted from the collected vaginal fluid, and the 16S rDNA in the V3-V4 regions was sequenced using the MiSeq System (Illumina, Inc., San Diego, CA, USA). Using the registered microbial identification database for type strains of bacterial species, the sequencing results for bacterial species that exhibited a homology rate of 97% or higher, along with the sequence that showed the highest homology rate, were presented. This method enables the identification of vaginal lactobacilli at the species level. The DNA extraction, sequencing of the 16S rDNA, and subsequent analysis of the results were conducted by TechnoSuruga Laboratory Co., Ltd. (Shizuoka, Japan).

### 2.4 Culture of vaginal fluid bacteria

After preparing a 10-fold serial dilution series of the bacterial suspension in Dulbecco’s phosphate-buffered saline (DPBS), 100 μL of each dilution was plated on different agar plates. The culture media used here were Lactobacilli MRS (Becton Dickinson and Co., Sparks, MD, USA), a selective culture medium designed for the effective growth of lactobacilli including *L. crispatus*, *L. gasseri*, and *L. jensenii*, GAM (Nissui Pharmaceutical Co., Ltd., Tokyo, Japan) containing 1% glucose, which is a non-selective medium for anaerobic bacteria, Tryptic Soy (Becton Dickinson and Co.) containing 5% sheep-defibrinated blood, which is a partially heat-inactivated, non-selective medium for *L. iners* and BV-associated bacteria, and Sabouraud agar plates (Nissui Pharmaceutical Co., Ltd.), which were used to cultivate *Candida* species. The MRS, GAM, and Tryptic Soy agar plates were incubated anaerobically in an anaeropack (Mitsubishi Gas Chemical Co., Inc., Tokyo, Japan) at 37°C for 5 days. Sabouraud agar plates were incubated aerobically at 28°C for 5 days. Then, bacterial colonies identical in size, color, and shape were determined, and viable counts (colony-forming units/mL, CFU/mL) were quantified by enumeration of the number of identical colonies. The number of viable bacteria from each appropriate culture medium was combined and plotted on a single graph.

### 2.5 Colony PCR

The obtained colonies were harvested and suspended in 30 µL of sterile nuclease-free water. Colony PCR was performed using 1 µL of the suspension as a template with the primers listed in **Supplementary Table 1** and KOD Fx Neo (TOYOBO Co., Ltd., Osaka, Japan) to identify the microbial genus or group (20 µL total, one cycle at 98°C for 10 min, 35 cycles at 98°C for 30 s, 60°C for 30 s, 72°C for 1 min, and one cycle at 72°C for 5 min).

### 2.6 Statistical analysis

Statistical analyses were performed using GraphPad Prism version 10.2.3 (GraphPad Software Inc., San Diego, CA, USA). To assess differences or correlations between the groups, we performed either the D’Agostino–Pearson omnibus normality test (the number of samples; n ≥ 8) or the Shapiro–Wilk normality test (n < 8) to determine whether to use parametric or nonparametric statistics (D’Agostino, 1986; Royston, 1995). We conducted an unpaired Student’s *t*-test or a Pearson’s correlation for parametric data and Mann-Whitney U-test or Spearman’s correlation for nonparametric data (Holder et al., 2018; Zhang et al., 2018; Chahal et al., 2022; Niemiec et al., 2022; Alfituri et al., 2024).

## 3 Results

### 3.1 Participant background information

Basic information regarding the participants is presented in **Supplementary Table 2**. All participants in this study were Japanese. These included women who had taken antibiotics within the past month (IDs 105, 120, 124, and 129), and those with female genital diseases (IDs 104 and 125). Therefore, six participants were excluded from the study because they did not meet the selection criteria. Two participants (IDs 116 and 126) were in their menstrual cycle and two (IDs 112 and 113) were pregnant. One participant (ID 109) was being administered gonadotropin-releasing hormone (GnRH) inhibitors. The analysis was conducted without exclusion criteria.

### 3.2 Analysis of vaginal microbiota

**Figure 1:**
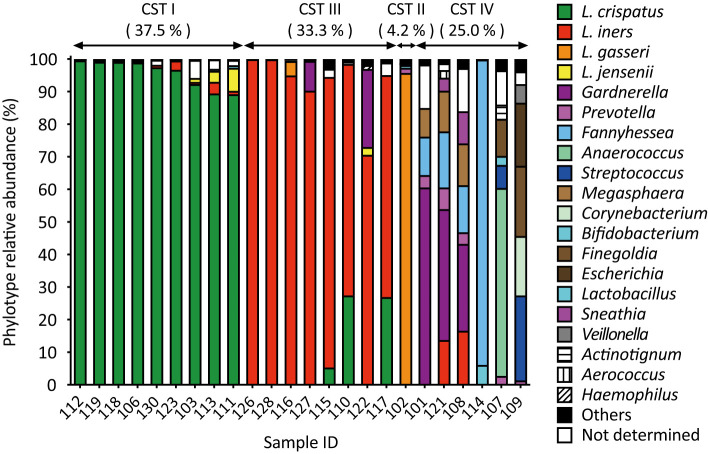
Amplicon sequencing analysis of vaginal fluid revealed that the vaginal microbiota of healthy Japanese women was categorized into four groups: *L. crispatus*, *L. iners*, *L. gasseri*, and a diversity group. Vaginal fluid was collected from 24 participants using a Copan eSwab device. Amplicon sequencing targeting the V3-V4 regions of 16S rDNA was conducted to identify the bacterial species present in the vaginal microbiota. Bacteria with a detection rate of 1% or higher are shown on the right side of the figure, whereas those with a 1% or less are classified as “others”.

Amplicon sequencing analysis revealed that *L. crispatus* (CST I) was the most abundant vaginal *Lactobacillus* species in 9 out of 24 samples (37.5%, **Figure 1**). *Lactobacillus iners* (CST III) was abundant in eight samples (33.3%) and *L. gasseri* (CST II) in one sample alone (4.2%, **Figure 1**). In addition to lactobacilli, *Gardnerella* was the next most abundant vaginal bacterium in three samples, followed by *Fannyhessea*, *Anaerococcus*, and *Streptococcus* in one sample each (CST IV: 25.0%, **Figure 1**).

**Figure 2:**
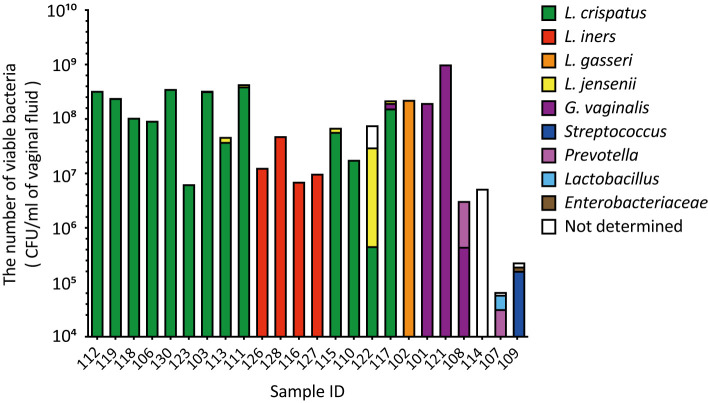
The viable counts in vaginal fluid obtained using culture methods varied significantly among the participants. Vaginal fluid was collected from 24 participants using a Copan eSwab device. Suspensions of the stored samples were plated on various agar plates and incubated for specific durations. The number of viable bacterial cells was calculated by counting the colonies per species. Colony PCR was performed using 16S rDNA as the target to identify the bacterial species in the vaginal microbiota. CFU, colony-forming unit.

In the combined culture and colony PCR analyses, *L. crispatus* was identified as the most abundant vaginal bacterial species in 13 samples (54.1%, **Figure 2**). *Lactobacillus iners* was the second most abundant vaginal bacterium in four samples (16.7%), followed by *G. vaginalis* in three samples (12.5%), and *L. gasseri*, *Streptococcus*, and *Prevotella* in one sample each (4.2%, **Figure 2**). The most abundant vaginal bacteria could not be identified in one sample (ID 114, **Figure 2**).

A comparison of the results of the combined culture and colony PCR analyses with those of amplicon sequencing showed that 18 of the 24 samples (75.0%) had the same results for the dominant vaginal bacterial populations (**Figures 1**, **2**). In four out of six samples (IDs 110, 115, 117, and 122), the most dominant bacteria identified differed depending on the analysis method; *L. iners* was identified as the most dominant bacteria using amplicon sequencing analysis (**Figure 1**). In the remaining two samples, *Fannyhessea* and *Anaerococcus*, which were not included in the target bacteria for colony PCR, were identified as the most abundant bacteria using amplicon sequencing analysis in sample IDs 107 and 114, respectively (**Figure 1**).

### 3.3 The number of viable bacteria in vaginal fluid

The number of viable bacteria in the vaginal fluid was calculated as the sum of individual viable bacteria with an average of 1.3 × 10^8^ CFU/mL (**Figure 2**). The highest viable count in the vaginal fluid was 1.0 × 10^9^ CFU/m: (ID 121). In contrast, the lowest count was 3.2 × 10^4^ CFU/mL (ID 107), with *Gardnerella* and *Anaerococcus* identified as the most dominant bacteria, respectively (**Figures 1**, **2**). The difference in the total number of viable bacteria between the most and least abundant samples was 3.1 × 10^4^ (**Figure 2**). The total number of viable lactobacilli in the *Lactobacillus-*dominated microbiota was more than 6.3 × 10^6^ CFU/mL in the vaginal fluid (**Figure 2**). In the *L. crispatus-*dominated samples, the highest viable count in the vaginal fluid was 4.3 × 10^8^ CFU/mL (ID 111), whereas the lowest count was 6.3 × 10^6^ CFU/mL (ID 123), representing a difference of more than 70-fold (**Figure 2**).

### 3.4 Associations among vaginal pH, relative *Lactobacillus* abundance, and the number of viable bacteria in vaginal fluid

**Figure 3:**
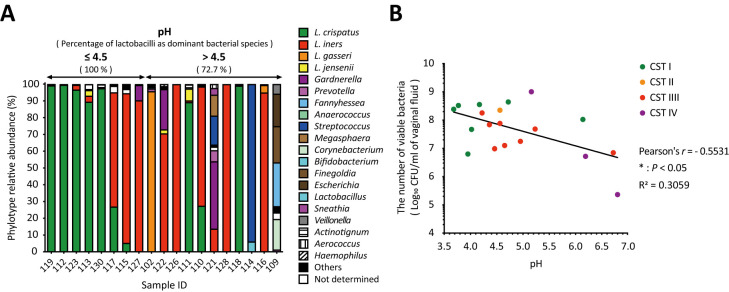
Negative correlation between vaginal pH and the number of viable bacteria in vaginal fluid. The relationship among vaginal pH, relative *Lactobacillus* abundance, and the number of viable bacteria in vaginal fluid was examined in 19 samples. **(A)** Results of vaginal microbiota composition using amplicon sequencing analysis were sorted from left to right by vaginal pH. **(B)** Correlation analysis was performed to calculate the Pearson’s correlation coefficient after performing the D’Agostino–Pearson omnibus normality test, and *P < 0.05 indicates statistically significant differences. The green, orange, red, and purple dots represent the dominant bacterial species classified as community state types: CST1, CST2, CST3, and CST4. CFU, colony-forming unit; CST, community state type.

The predominant species in the pH ≤ 4.5 group were all lactobacilli, whereas the BV-associated bacteria detected as the predominant species were all in the pH above 4.5 group (**Figure 3A**). Lactobacilli were more than 90% abundant in the pH ≤ 4.5 group and the five samples with the lowest pH were dominated by *L. crispatus* (**Figure 3A**). Vaginal pH was found to have a statistically significant negative correlation with the total number of viable bacteria in vaginal fluid (Pearson’s *r* = -0.5531, * *P* < 0.05; **Figure 3B**) across all groups.

### 3.5 Associations among BMI, relative *Lactobacillus* abundance, and the number of viable bacteria in vaginal fluid

**Figure 4:**
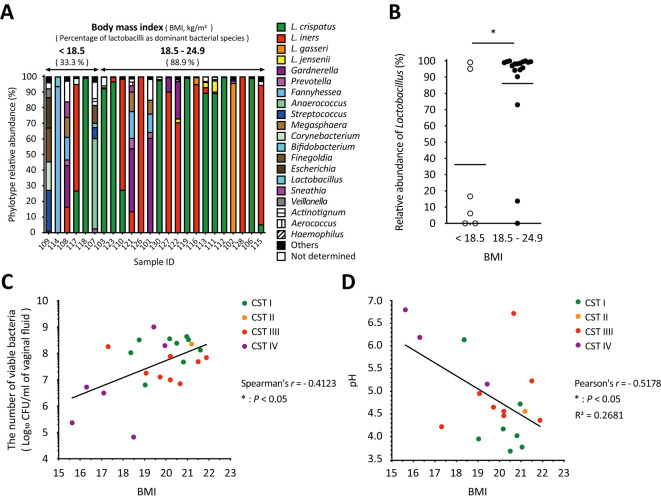
Body mass index significantly influenced vaginal microbiota, the number of viable bacteria, and vaginal pH in vaginal swabs. **(A)** Results of the vaginal microbiota composition analyzed with amplicon sequencing were sorted by BMI from left to right. **(B)** The relative abundance of *Lactobacillus* in each sample with a BMI of <18.5 kg/m^2^ (underweight) or 18.5–24.9 kg/m^2^ (normal weight) was plotted. Statistical analysis was performed using the Mann–Whitney U-test, and *P < 0.05 indicates a statistically significant difference. The association between **(C)** the number of viable bacteria in the vaginal fluid or **(D)** vaginal pH and BMI is highlighted. The correlation was evaluated by calculating the **(C)** Spearman’s or **(D)** Pearson’s correlation coefficient after performing the D’Agostino–Pearson omnibus normality test, and *P < 0.05 indicates statistically significant differences. The green, orange, red, and purple dots indicate the dominant bacterial species classified as community state types: CST1, CST2, CST3, and CST4. BMI, body mass index; CFU, colony-forming unit; CST, community state type.

As shown in **Figure 4A**, the amplicon sequencing analysis classified the vaginal microbiota into two groups: six samples from participants with a BMI of less than 18.5 kg/m^2^, classified as underweight by the World Health Organization (WHO), and eighteen samples from participants with a BMI of 18.5–24.9 kg/m^2^, classified as normal weight. The underweight group, which had no health issue problems and no special characteristics, had a lower proportion of women with *Lactobacillus* as the dominant species compared to the normal weight group (**Figure 4A**). In addition, the relative abundance of *Lactobacillus* in the underweight group was statistically significantly lower than that in the normal weight group (**Figure 4B**). Moreover, correlation analyses revealed a statistically significant positive correlation between BMI and total viable bacterial count (Spearman’s *r* = 0.4123, * *P* < 0.05; **Figure 4C**) and a statistically significant negative correlation with vaginal pH (Pearson’s *r* = -0.5178, * *P* < 0.05; **Figure 4D**) across all groups. No correlation was found between BMI and dominant bacterial species (**Figure 4**).

### 3.6 Association among age, relative *Lactobacillus* abundance, the number of viable bacteria counts, and vaginal fluid pH

**Figure 5:**
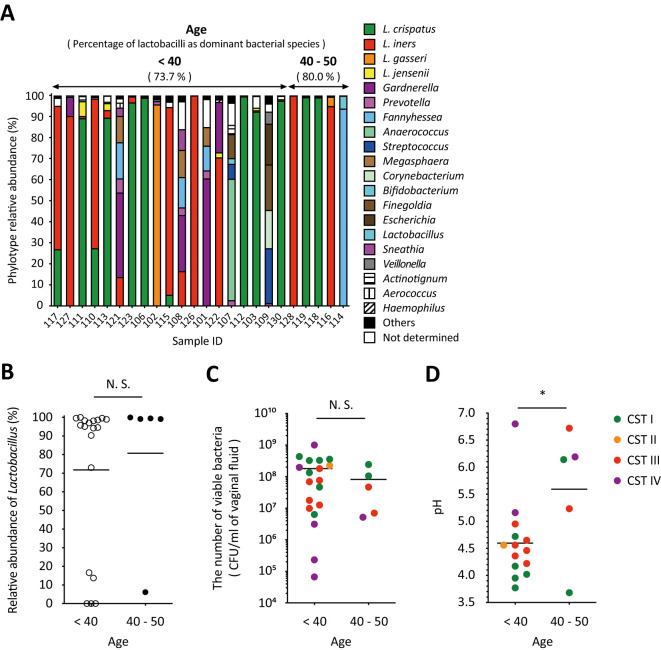
The vaginal pH of women aged 40 to 50 was significantly higher than that of women under 40. **(A)** Results of vaginal microbiota composition using amplicon sequencing analysis were sorted by age from left to right. **(B)** The relative abundance of *Lactobacillus* in each sample in women <40 or 40–50 years of age was plotted. The association between **(C)** the number of viable bacteria in vaginal fluid or **(D)** vaginal pH and age (women <40 or 40–50 years old) is highlighted. Statistically significant differences *P < 0.05 are indicated based on the results of the unpaired *t*-test. The green, orange, red, and purple dots indicate the dominant bacterial species classified as community state types: CST1, CST2, CST3, and CST4. CFU, colony-forming unit; N.S., not significant; CST, community state type.

All participants were premenopausal women. The proportion of participants with *Lactobacillus* as the dominant species and the relative abundance of *Lactobacillus* in each sample did not show significant differences between the <40- and 40–50-year-old groups (**Figures 5A, B**). The total viable bacterial count was also not significantly different between the groups (**Figure 5C**). In contrast, the vaginal pH of women aged <40 years was statistically significantly lower than that of women in the 40–50-year-old group (**Figure 5D**). No correlation was found between age and dominant bacterial species (**Figure 5**).

### 3.7 Association among the use of COC or hormonal cycle, relative *Lactobacillus* abundance, the number of viable bacteria counts, and vaginal fluid pH

The proportion of participants with *Lactobacillus* as the dominant species and the relative abundance of *Lactobacillus* in each sample did not show statistically significant differences between the COC non-user and user groups (**Supplementary Figures 1A, B**). The total viable bacterial count and vaginal pH showed no statistically significant differences between the groups (**Supplementary Figures 1C, D**). In addition, the proportion of participants with *Lactobacillus* as the dominant species, as well as the relative abundance of *Lactobacillus* in each sample, did not show statistically significant differences among the groups categorized by days since the first day of the last menstrual period (1-14 days, 15-28 days, and 29 days or more) (**Supplementary Figures 2A, B**). The total viable bacterial count and vaginal pH also did not show statistically significant differences among the groups (**Supplementary Figures 2C, D**).

## 4 Discussion

### 4.1 Main findings

In this study, we analyzed the vaginal microbiota of healthy Japanese women using amplicon sequencing and a combination of culture and colony PCR techniques. The vaginal microbiota of the participants was classified into four types: *Lactobacillus crispatus* (CST I, 37.5%), *Lactobacillus iners* (CST III, 33.3%), *Lactobacillus gasseri* (CST II, 4.2%), and a diversity group (CST IV, 25.0%) (**Figure 1**). Interestingly, the most dominant bacterial species identified were consistent across both methods (**Figures 1**, **2**), whereas the viable bacterial counts obtained by the culture methods varied significantly among the participants (**Figure 2**). This is the first study to demonstrate such wide variation in viable vaginal bacteria using a combination of quantitative and relative analyses. Additionally, we found that total viable bacterial counts were negatively correlated with vaginal pH (**Figure 3**). The results showed a statistically significant association between body mass index (BMI) and both vaginal viable bacterial count and vaginal pH (**Figure 4**). Furthermore, a relationship between age and vaginal pH was observed (**Figure 5**). Our findings indicate that the vaginal microbiome of healthy Japanese women is not only highly diverse but also affected by host factors such as BMI and age.

### 4.2 Interpretation

The analysis of the composition of the vaginal microbiota using metagenomics is a widely used method; however, it has certain limitations, including a lack of information about bacterial viability. Therefore, if combined with molecular and cultural methods, they can provide a much more comprehensive understanding of the vaginal microbiota and address the unknown aspects of metagenomic studies. *Lactobacillus* is identified as the most predominant bacterial species, including *L. crispatus*, *L. iners*, *L. gasseri*, and *L. jensenii* in normal vaginal microbiota (Ravel et al., 2011). Low levels of several species were also detected in the healthy vaginal microbiota, such as *Gardnerella*, *Prevotella*, *Staphylococcus, Streptococcus*, *Escherichia*, *Fannyhessea*, and *Mobiluncus* (Ravel et al., 2011; Gajer et al., 2012; Kurakawa et al., 2015). Some bacteria associated with BV and AV could act as opportunistic pathogens (Onderdonk et al., 2016; Chacra et al., 2022; Jahic, 2022). To isolate possible bacteria from the healthy vaginal microbiota by conventional culture methods, different bacterial growth conditions (anaerobic and aerobic) and culture media (selective and non-selective) were used along with bacteria with individual characteristics. The vaginal environment was reported to have average oxygen levels ranging from 15 to 35 mmHg (2-5%) and carbon dioxide levels from 35 to 55 mmHg (5-7%), indicating a microaerophilic condition (Wagner et al., 1984; Hill et al., 2005; O’Hanlon et al., 2013; Tachedjian et al., 2018). On the other hand, most vaginal bacteria prefer to be cultured under anaerobic conditions; therefore, we cultured them under anaerobic conditions using the AnaeroPack gas-pack system, which generates carbon dioxide and provides anaerobic conditions (oxygen levels remain at 0.1% or lower). In this method, anaerobic bacteria associated with BV, such as *Gardnerella* and *Prevotella*, as well as aerobic bacteria associated with AV, including *Escherichia* and *Streptococcus*, were successfully cultured and detected (**Figure 2**), showing that these culture conditions are sufficient to cultivate a low number of vaginal bacteria. Vaginal fluid was cultured on four types of selective media: Lactobacilli MRS, GAM with 1% glucose, Tryptic Soy with 5% defibrinated sheep blood, and Sabouraud agar plates, following ATCC, BEI resources, or the National Institute of Technology and Evaluation (NITE), Japan handling recommendations and previous reports on vaginal bacteria (Matsuda et al., 2007, 2009; Kurakawa et al., 2015; Łaniewski et al., 2017; Diallo et al., 2018; Ilhan et al., 2020; McKenzie et al., 2021; Maarsingh et al., 2022). BV-associated bacteria, including *Gardnerella*, *Prevotella*, *Fannyhessea*, *Mobiluncus*, *Anaerococcus*, *Peptostreptococcus*, and various gut bacteria, including *Escherichia* and *Bifidobacterium*, were reported to be culturable in or on GAM using a broth or agar plate containing 1% glucose (Matsuda et al., 2007, 2009; Kurakawa et al., 2015). Tryptic Soy Broth was demonstrated to be the most effective among the 11 media tested for culturing vaginal bacteria from vaginal fluid under anaerobic conditions, resulting in the cultivation of 46 bacterial species (Chacra et al., 2024). Tryptic Soy Medium with 5% defibrinated sheep blood has been shown to effectively cultivate BV and urogenital infection-associated bacteria, including *Gardnerella*, *Prevotella*, *Fannyhessea*, *Mobiluncus*, *Neisseria*, *Fusobacterium*, *Eggerthella*, *Peptoniphilus*, *Porphyromonas*, and *Actinotignum* (Matsuda et al., 2007; Kurakawa et al., 2015; Łaniewski et al., 2017; Diallo et al., 2018; Ilhan et al., 2020; McKenzie et al., 2021; Maarsingh et al., 2022). It is also recommended by ATCC, BEI resources, or NITE for cultivating *Staphylococcus*, *Streptococcus*, *Megasphaera*, *Corynebacterium*, *Finegoldia*, *Veillonella*, and *Aerococcus*. Additionally, heat-inactivation to inhibit Factor V degradation in sheep blood aids in cultivating *Haemophilus* and *Sneathia* because Factor V in the blood is essential for cultivation (Rennie et al., 1992; Hanff et al., 1995; Harwich et al., 2012). However, culturing *Chlamydia* and *Treponema pallidum* using these methods is believed to be challenging. Consequently, viable bacteria described here indicate that not all bacteria but culturable bacteria grown under different media and growth conditions. Nevertheless, since unculturable bacteria such as *Chlamydia* or *Treponema* were not detected in the 16S rDNA results (**Figure 1**), this limitation does not affect the validity of our current conclusions.

To the best of our knowledge, this is the first study to report the differences in viable bacterial counts in vaginal fluid samples. Viable counts varied widely among the participants, even when the most dominant vaginal bacteria were identified as the same species (**Figure 2**). Compared with the gut microbiota, where 10-fold differences in the microbial loads of healthy individuals have been observed (Vandeputte et al., 2017), the differences in the number of viable bacteria in the vaginal fluid were high (**Figure 2**). Reverse transcription-quantitative PCR (RT-qPCR), which targets microbial rRNA molecules generally found in living bacteria alone, quantifies the predominant human gut bacteria with a sensitivity equivalent to that of qPCR or fluorescence *in situ* hybridization (FISH), and reveals several interesting quantitative differences in the gut microbiota of individuals (Matsuda et al., 2007, 2009, 2012; Kubota et al., 2010; Kurakawa et al., 2012, 2013). Analysis of vaginal microbiota in healthy Japanese women using RT-qPCR showed that the total number of lactobacilli in the *Lactobacillus*-dominant microbiota was found to be more than 10^7^ cells/mL. In contrast, the highest population level of *G. vaginalis* was found to be 10^8.8^ cells/mL in the vaginal fluid (Kurakawa et al., 2015). In this study, we demonstrated that the number of viable bacteria in the vagina determined using the culture method closely aligned with the results of RT-qPCR analysis, highlighting the accuracy of the culture method in quantifying the number of viable vaginal bacteria.

In previous studies, analysis of the vaginal microbiota of healthy Japanese women using amplicon sequencing revealed that the most prevalent vaginal microbiota was *L. crispatus* (40.2%–50.0%), followed by *L. iners* (25.0–27.8%) (Zhou et al., 2010; Kurakawa et al., 2015). Similar results were obtained in this study, showing that *L. crispatus* was the most abundant vaginal bacterial species (37.5%; **Figure 1**), followed by *L. iners* (33.3%; **Figure 1**). Differences among the culture, colony PCR analysis, and amplicon sequencing results were mainly due to the detection rate of *L. iners* (**Figures 1**, **2**). This could be attributed to the presence of *L. iners* in a viable but non-culturable (VBNC) state in the vagina and/or the detection of dead *L. iners* by next-generation sequencing but not by the culture method. In addition, the combined culture and colony PCR method did not identify *Fannyhessea* and *Anaerococcus* because they were not targets for PCR analysis (**Figure 2**). Although the culture method could accurately identify the most common bacteria, for more precise analysis, it is necessary to conduct further investigations using additional primer sets, including those for BV-associated bacteria, such as *Fannyhessea* (Liu et al., 2023), *Anaerococcus* (Africa et al., 2014)*, Sneathia* (Srinivasan et al., 2012), and *Megasphaera* (Glascock et al., 2021).

Most reports of vaginal pH have been measured using pH indicator paper. This is an easier, cheaper, and quicker method of obtaining a pH value; however, the pH value is determined by comparison to the color scale, which indicates pH ranges rather than specific values, with increments typically ranging from 0.3 to 1.0 on the pH scale. To obtain a more accurate vaginal pH value and minimize measurement errors, using a pH meter is preferable. In this study, vaginal pH was measured using a pH meter with sampling paper that requires only 50 µL of vaginal fluid and allows for measurements up to two decimal places. The pH of the lactobacilli*-*dominant microbiota in the vaginal fluid was 4.5 or less (Mirmonsef et al., 2014; Spear et al., 2015), whereas a decrease in lactobacilli dominance is associated with BV, which is characterized by an overgrowth of anaerobic bacteria and a vaginal pH of above 4.5 (Aldunate et al., 2015). Consistent with the previous reports (Mirmonsef et al., 2014; Spear et al., 2015), our study revealed that the predominant species in the group with pH 4.5 or less were exclusively lactobacilli, and they accounted for more than 90% of the species abundance, but not in the group with the pH >4.5 that was predominant with BV-associated bacteria (**Figure 3A**). Interestingly, we found a statistically significant negative correlation between vaginal pH and the total number of viable bacteria in vaginal fluid (**Figure 3B**). This indicates that the vaginal pH is associated with the total viable count of vaginal bacteria.

Several factors, such as BMI and age, had a significant impact on the vaginal microbiome (**Figures 4**, **5**). Women with a BMI greater than 25 kg/m^2^, who are classified as overweight, are more likely to have a higher prevalence of BV-associated bacteria, such as *Gardnerella* and *Prevotella*, in the vaginal microbiota (Brookheart et al., 2019; Raglan et al., 2021; Allen et al., 2022). In addition to the outcome of being overweight, a similar effect was observed in underweight women (Brookheart et al., 2019), indicating that BMI influences the vaginal microbiota. Our results showed that the abundance of *Lactobacillus* in normal-weight women (BMI of 18.5–24.9 kg/m^2^) was statistically significantly higher than that in underweight women (BMI less than 18.5 kg/m^2^, **Figure 4B**), suggesting that BMI is associated with the relative abundance of *Lactobacillus*. Additionally, there was a statistically significant positive correlation between BMI and viable bacterial counts in the vagina, as well as a statistically significant negative correlation with vaginal pH (**Figures 4C, D**). However, all BMI values in this study were below 22 kg/m^2^ (**Figures 4C, D**). These results indicate that the BMI is associated with total vaginal viable count and vaginal pH. Specifically, our study showed the possibility that being underweight could lead to a less stable vaginal microbiota by reducing the relative abundance of *Lactobacillus*, the total vaginal viable bacterial counts, and increasing the vaginal pH (**Figure 4**).

In addition to BMI, age has been shown to influence the vaginal microbiota (Wang et al., 2021). Wang et al. showed that age has a significant positive correlation with microbial diversity (Wang et al., 2021). Average Shannon diversities were relatively stable in women under 40 years of age; however, it began to fluctuate in women aged >40 years, suggesting that the period between 40 and 50 years may be a critical stage in which the vaginal microbiome becomes more fragile and vulnerable to dysbiosis (Wang et al., 2021). Our results showed that the relative abundance of *Lactobacillus* and the total vaginal viable counts in individuals under 40 years of age were almost the same as those in individuals between 40 and 50 years of age (**Figures 5B, C**). This finding indicates that age does not affect either the *Lactobacillus* abundance or the total vaginal viable counts. However, the vaginal pH of women aged 40–50 years was statistically significantly higher than that of women under 40 years, even though the proportion of *Lactobacillus* as the dominant bacterial species was similar in both age groups (**Figures 5A, D**). These results suggest that women aged 40–50 years have a significant effect on vaginal pH without undergoing a notable change in the total number of viable vaginal bacteria, reflecting perimenopausal status. All samples with above-average pH from individuals over the age of 40 were collected from participants aged 45 and older, although one participant was taking oral contraceptives (**Figure 5D**). A portion of women aged 40–50 is classified as perimenopausal (Hale et al., 2014; McCarthy and Raval, 2020; Turek and Gąsior, 2023). During this phase, estrogen levels are known to periodically decline to postmenopausal levels (Burger et al., 2007; Gordon et al., 2016). It has also been noted that the decrease in estrogen during perimenopause results in an increase in vaginal pH (Delamater and Santoro, 2018), while Santoro et al. noted that understanding the biological mechanisms of perimenopause is complex (Santoro, 2016). Although the exact biological mechanisms that cause an increase in vaginal pH without a significant change in the total number of viable vaginal bacteria remain unclear, the vaginal pH value of women in this age group serves as a good indicator of the influence of age on the vaginal microbiome.

The use of combined oral contraceptives (COC) has been suggested to impact the vaginal microbiota (Bastianelli et al., 2021; Tuddenham et al., 2023). Bastianelli et al. indicated that oral contraceptives may have a beneficial effect on the vaginal microbiota composition since COC users tend to have significantly less diverse vaginal microbiota (Bastianelli et al., 2021). Tuddenham et al. also found that COC is associated with greater stability in vaginal microbiota, often resulting in a *Lactobacillus*-dominated state (Tuddenham et al., 2023). Conversely, several studies indicate no significant difference in bacterial composition between COC users and non-users (Donders et al., 2017; Song et al., 2020). In our study, no statistically significant differences were observed in the diversity of the vaginal microbiota or the relative abundance of *Lactobacillus* (**Supplementary Figures 1A, B**). Additionally, no statistically significant differences in viable vaginal bacterial counts or vaginal pH were observed between COC users and non-users (**Supplementary Figures 1C, D**). It has been suggested that the relationships between COC and *Lactobacillus* dominance, as well as the stability of the microbiota, vary by human race (Tuddenham et al., 2023). In addition, the authors provided data for two years of COC use (Tuddenham et al., 2023). However, this study lacks comprehensive information; therefore, further research is needed to clarify the effects of COC use on the vaginal microbiome.

During the hormonal cycle, estrogen levels rise during the proliferative phase, which spans from days 1 to 14 of the menstrual period, and decline during the secretory phase, which ranges from days 15 to 28 (Artym and Zimecki, 2021). It has been reported that there is no significant difference in the relative abundance of *Lactobacillus* between the proliferative and secretory phases (Cheng et al., 2021). In this study, the relative abundance of *Lactobacillus* decreased, while viable vaginal bacterial counts and vaginal pH increased during the secretory phase compared to the proliferative phase (**Supplementary Figures 2A–C**). However, these changes were not statistically significant (**Supplementary Figures 2A–C**). Samples collected 28 days or more after the first day of the last menstrual period showed that the viable vaginal bacterial count was nearly comparable to that observed during the secretory phase (**Supplementary Figure 2C**). However, the vaginal pH was 0.5 units lower (**Supplementary Figure 2D**). This difference is thought to result from the higher abundance of *Lactobacillus* compared to the secretory phase (**Supplementary Figure 2B**).

## 5 Conclusion

Based on the dominant bacterial populations, the vaginal microbiota of healthy adult Japanese women can be classified into four types. Moreover, our combined relative and quantitative analyses revealed individual differences in the total number of viable bacteria in vaginal fluid. We also found that the total viable bacteria were negatively correlated with vaginal pH. Additionally, factors such as BMI and age can influence the vaginal microbiome, indicating significant diversity in the vaginal microbiome among individuals. In conclusion, although our study does not address all existing biases in microbiome research, we propose a simple and easily implementable method to quantitatively assess variations in the vaginal microbiota. In the future, vaginal microbiome studies based on both relative and quantitative analyses are needed to accurately characterize host-microbiota interactions.

### 5.1 Strengths and limitations

The main strength of this study is the novel combination of quantitative and relative analyses to assess the diversity and viability of the vaginal microbiota. Using a pH meter instead of pH indicator paper allowed us to more effectively examine the relationship between pH levels, microbiota composition, viable bacterial counts, and various host factors. These approaches enabled a more comprehensive understanding of the vaginal microbiome and its relationship with host factors. Here, we demonstrated the diversity of vaginal microbiota in healthy Japanese women. However, our study has several limitations. First, the number of participants in this study was limited. Therefore, we need to verify our findings in a larger study. Second, some bacterial species, including *Chlamydia trachomatis* and *Treponema pallidum*, cannot be cultured by the culture method used in this study; notably, these species were not detected by amplicon sequencing analysis. Therefore, alternative quantification methods, such as RT-qPCR (Kurakawa et al., 2015), are required to detect and quantify viable bacteria.

## Data Availability

The original contributions presented in the study are included in the article/**Supplementary Material**. Further inquiries can be directed to the corresponding author.
