# Supplementary material for: Diverse vaginal microbiota in healthy Japanese women: a combined relative and quantitative analyses
Source: Front Cell Infect Microbiol. 2025 Feb 4;14:1487990. doi: 10.3389/fcimb.2024.1487990 (PMC11832463; doi:10.3389/fcimb.2024.1487990)
Supplement: Supplementary file 1 [file DataSheet1.pdf]

Supplementary Material

Supplementary Figure Legends

Supplementary Figure 1. The use of combined oral contraceptives did not significantly influence vaginal microbiota, the number of viable bacteria, or vaginal pH in vaginal swabs.

(A) Results of vaginal microbiota composition using amplicon sequencing analysis were sorted based on the non-use or use of combined oral contraceptives (COC). (B) The relative abundance of *Lactobacillus* in each sample, categorized by the non-use or use of COC, was plotted. Statistical analysis was performed using the Mann–Whitney U-test. The association between (C) the number of viable bacteria in the vaginal fluid or (D) vaginal pH and the use of COC is highlighted. Statistical analysis was also performed using the Mann–Whitney U-test. The green, orange, red, and purple dots represent the dominant bacterial species classified as community state types: CST1, CST2, CST3, and CST4. Abbreviations: COC, combined oral contraceptives; CFU, colony-forming unit; CST, community state type.

Supplementary Figure 2. The hormonal cycle did not significantly influence vaginal microbiota, the number of viable bacteria, or vaginal pH in vaginal swabs.

(A) Results of vaginal microbiota composition using amplicon sequencing analysis were sorted by the days since the first day of the last menstrual period from left to right. (B) The relative abundance of *Lactobacillus* in each sample, categorized by the days since the first day of the last menstrual period (1-14, 15-28, and 28 or more), was plotted. Statistical analysis was performed using the Mann–Whitney U-test. The association between (C) the number of viable bacteria in the vaginal fluid or (D) vaginal pH and the use of COC is highlighted. Statistical analysis was also performed using the Mann–Whitney U-test. The green, orange, red, and purple dots represent the dominant bacterial species classified as community state types: CST1, CST2, CST3, and CST4. Abbreviations: CFU, colony-forming unit; CST, community state type.

Supplementary References

Backer, E. D., Verhelst, R., Verstraelen, H., Alqumber, M. A., Burton, J. P., Tagg, J. R., et al. (2007). Quantitative determination by real-time PCR of four vaginal *Lactobacillus* species, *Gardnerella vaginalis* and *Atopobium vaginae* indicates an inverse relationship between *L. gasseri* and *L. iners*. *BMC Microbiol.* 7, 115. doi: 10.1186/1471-2180-7-115

Byun, R., Nadkarni, M. A., Chhour, K.-L., Martin, F. E., Jacques, N. A., and Hunter, N. (2004). Quantitative analysis of diverse *Lactobacillus* species present in advanced dental caries. *J. Clin. Microbiol.* 42, 3128–3136. doi: 10.1128/jcm.42.7.3128-3136.2004

Farrell, D. J. (1999). Evaluation of AMPLICOR *Neisseria gonorrhoeae* PCR using *cppB* nested PCR and 16S rRNA PCR. *J. Clin. Microbiol.* 37, 386–390. doi: 10.1128/jcm.37.2.386-390.1999

Kurakawa, T., Ogata, K., Tsuji, H., Kado, Y., Takahashi, T., Kida, Y., et al. (2015). Establishment of a sensitive system for analysis of human vaginal microbiota on the basis of rRNA-targeted reverse transcription-quantitative PCR. *J. Microbiol. Methods* 111, 93–104. doi: 10.1016/j.mimet.2015.01.021

Matsuda, K., Tsuji, H., Asahara, T., Kado, Y., and Nomoto, K. (2007). Sensitive quantitative detection of commensal bacteria by rRNA-targeted reverse transcription-PCR. *Appl. Environ. Microbiol.* 73, 32–39. doi: 10.1128/aem.01224-06

Matsuda, K., Tsuji, H., Asahara, T., Matsumoto, K., Takada, T., and Nomoto, K. (2009). Establishment of an analytical system for the human fecal microbiota, based on reverse transcription-quantitative PCR targeting of multicopy rRNA molecules. *Appl. Environ. Microbiol.* 75, 1961–1969. doi: 10.1128/aem.01843-08

Matsuki, T., Watanabe, K., Fujimoto, J., Miyamoto, Y., Takada, T., Matsumoto, K., et al. (2002). Development of 16S rRNA-gene-targeted group-specific primers for the detection and identification of predominant bacteria in human feces. *Appl. Environ. Microbiol.* 68, 5445–5451. doi: 10.1128/aem.68.11.5445-5451.2002

Ogata, K., Matsuda, K., Tsuji, H., and Nomoto, K. (2015). Sensitive and rapid RT-qPCR quantification of pathogenic *Candida* species in human blood. *J. Microbiol. Methods* 117, 128–135. doi: 10.1016/j.mimet.2015.07.021

Tamrakar, R., Yamada, T., Furuta, I., Cho, K., Morikawa, M., Yamada, H., et al. (2007). Association between *Lactobacillus* species and bacterial vaginosis-related bacteria, and bacterial vaginosis scores in pregnant Japanese women. *BMC Infect. Dis.* 7, 128. doi: 10.1186/1471-2334-7-128

Supplementary Table 1. Primers used in this study.

Target	Primer	Primer sequence (5'-3')	Product length (bp)	Reference
L. crispatus	s-Lcri-F	ATGTGTTTTAAAGAGCACGTGG	570	(Kurakawa et al., 2015)
	s-Lcri-R	GGCTAACCAATCTCTTGGCT		
L. iners	InersFw	GTCTGCCTTGAAGATCGG	158	(Backer et al., 2007)
	InersRev	ACAGTTGATAGGCATCATC		
L. gasseri	s-Lgas-F	GATAACAACACTAGACGCATGT	301	(Backer et al., 2007; Kurakawa et al., 2015)
	LgassR	CAGTTACTACCTCTATCTTTCTTCACTAC		
L. jensenii	LjensF	AAGTCGAGCGAGCTTGCCTATAGA	172	(Backer et al., 2007; Tamrakar et al., 2007)
	LjensR	ACGCCGCCTTTTAAACTTCTT		
Lactobacillus	LactoF	TGGAAACAGRTGCTAATACCG	231-233	(Byun et al., 2004)
	LactoR	GTCCATTGTGGAAGATTCCC		
G. vaginalis	s-Gvag-F	AGTGTGAACTTGTCGTGGAC	437	(Kurakawa et al., 2015)
	s-Gvag-R	ATCACCGGAATCAGCCTTACA		
Prevotella	g-Prevo-F	CACRGTAAACGATGGATGCC	527-529	(Matsuki et al., 2002)
	g-Prevo-R	GGTCGGGTTGCAGACC		
C. albicans	s-Calb-F	ATGTGGCACGGCTTCTGCTG	53	(Ogata et

	s-Calb-R	TAGGCTGGCAGTATCGTCAGAGG		al., 2015)
Streptococcus	g-Str-F<br>g-Str-R	AGCTTAGAAGCAGCTATTCATTC<br>GAGAGACCGAAAGGTGTATCC	309	(Kurakawa et al., 2015)
Staphylococcus	g-Staph-F<br>g-Staph-R	TTTGGGCTACACACGTGCTACAATGGACAA<br>AACAACTTTATGGGATTTGCWTGA	79	(Matsuda et al., 2009)
N. gonorrhoeae	Ngon_SL67-F<br>Ngon_SL59-R	TATCGGAACGTACCGGGTAGC<br>GTATTACCGCGGCTGCTGGCA	414	(Farrell, 1999)
M. curtisii	s-Mcur-F<br>s-Mcur-R	CCTAATGAGTGTGATAGCGTA<br>AACCCAGCACCATGCCAAAC	165	(Kurakawa et al., 2015)
Enterobacteriaceae	En-lsu-3F<br>En-lsu-3'R	TGCCGTAACTTCGGGAGAAGGCA<br>TCAAGGACCAGTGTTCAGTGTC	428	(Matsuda et al., 2007)
Enterococcus	g-Encoc-F<br>g-Encoc-R	ATCAGAGGGGGATAACACTT<br>ACTCTCATCCTTGTTCTTCTC	337	(Matsuda et al., 2009)
Bifidobacterium	g-Bifid-F<br>g-Bifid-R	CTCCTGGAAACGGGTGG<br>GGTGTTCTTCCCGATATCTACA	549-563	(Matsuki et al., 2002)

Supplementary Table 2. Baseline characteristics of participants in this study were characterized using vaginal swab samples.

Characteristic		All Participants (n = 24)		Correct pH measurement (n = 19)	
Age (years)		32.7 ±	7.8 (19-49)	32.9 ±	8.6 (19-49)
BMI		19.6 ±	1.7 (15.6-21.8)	19.7 ±	1.7 (15.6-21.8)
Temperature (°C)		36.3 ±	0.4	36.3 ±	0.5
Vaginal pH		4.9 ±	1.0 (3.68-6.80)	4.9 ±	1.0 (3.68-6.80)
Swab volume (mg)		239.5 ±	52.1	238.3 ±	51.8
Menstrual Cycle (day)		29.1 ±	4.4	28.9 ±	4.8
Menstrual Period (day)		5.6 ±	1.2	5.6 ±	1.2
Days since the start of last menstruation (day)		108.0 ±	289.7 (2-1,430)	131.0 ±	323.3 (2-1,430)
Current menstruation, n (%)		2	(8.3)	2	(8.3)
History of current illness, n (%)	Pseudohypoparathyroidism	1	(4.2)	1	(5.3)
Past history, n (%)	Chlamydial infection	2	(8.3)	1	(5.3)
	Herpes infection	1	(4.2)	1	(5.3)
	Cervical cancer	2	(8.3)	2	(10.5)
Taking medications, n (%)	Gonadotropin-releasing Hormone (GnRH) inhibitors	1	(4.2)	1	(4.2)
Use of combined oral contraceptives, n (%)		12	(50.0)	10	(52.6)
Vaginal washing routine, n (%)		0	(0.0)	0	(0.0)
Allergy, n (%)	Pollen	9	(37.5)	8	(42.1)
	House dust	4	(16.7)	3	(15.8)
	Animal hair	3	(12.5)	3	(15.8)
	Penicillin	1	(4.2)	1	(5.3)
Ever pregnant, n (%)		7	(29.2)	4	(21.1)
Current Pregnancy, n (%)		2	(8.3)	2	(10.5)
Childbirth Experience, n (%)		3	(12.5)	1	(5.3)
Delivery Method, n (%)	Vaginal delivery	2	(8.3)	1	(5.3)
	Cesarean section	0	(0.0)	0	(0.0)
History of spontaneous abortion, n (%)		1	(4.2)	0	(0.0)

History of abortion, n (%)	3	(12.5)	1	(5.3)
History of emergency contraceptive use, n (%)	1	(4.2)	0	(0.0)
