# Supplementary figures and images for: Diverse vaginal microbiota in healthy Japanese women: a combined relative and quantitative analyses
Source: Front Cell Infect Microbiol. 2025 Feb 4;14:1487990. doi: 10.3389/fcimb.2024.1487990 (PMC11832463; doi:10.3389/fcimb.2024.1487990)

**A**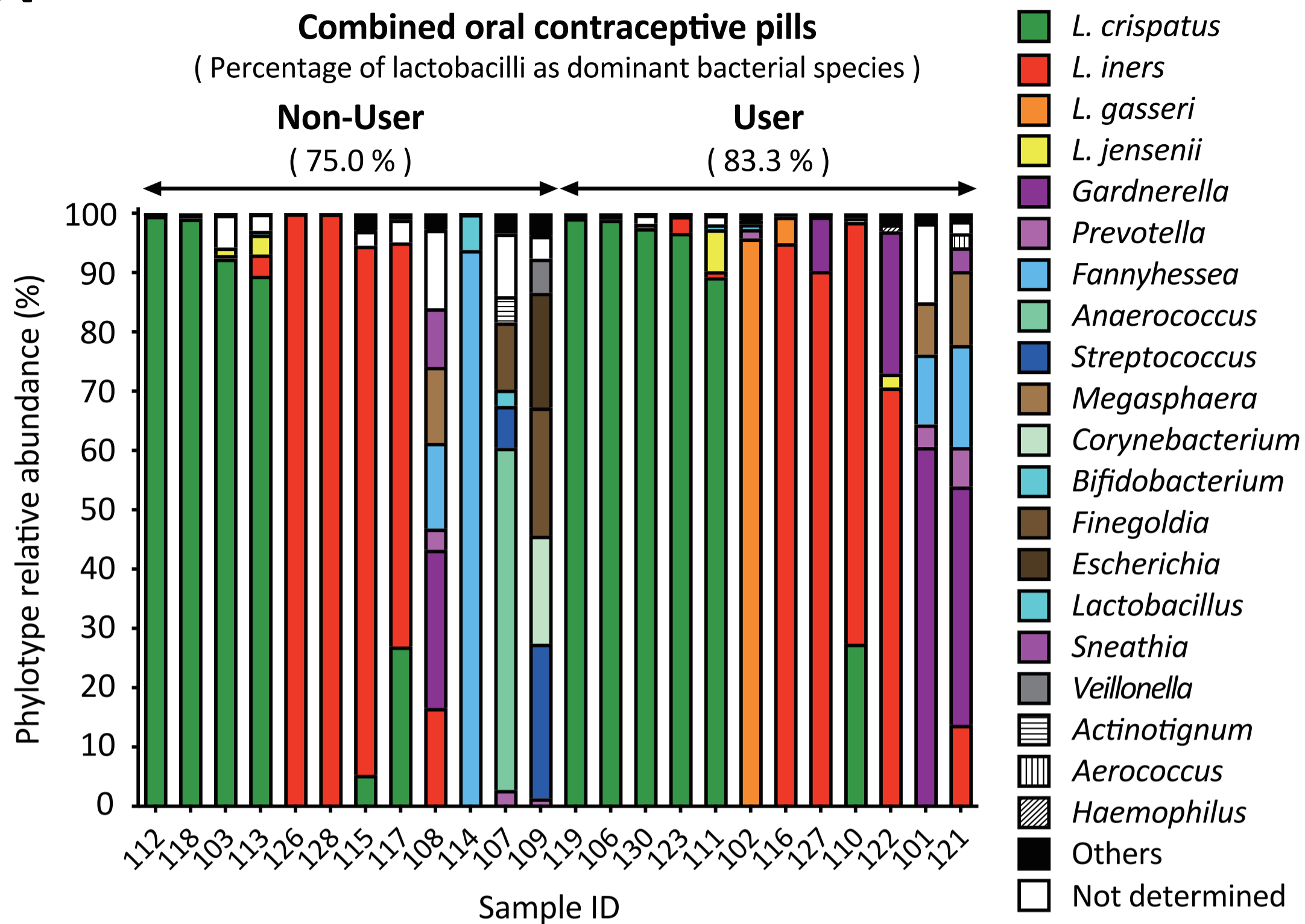**B**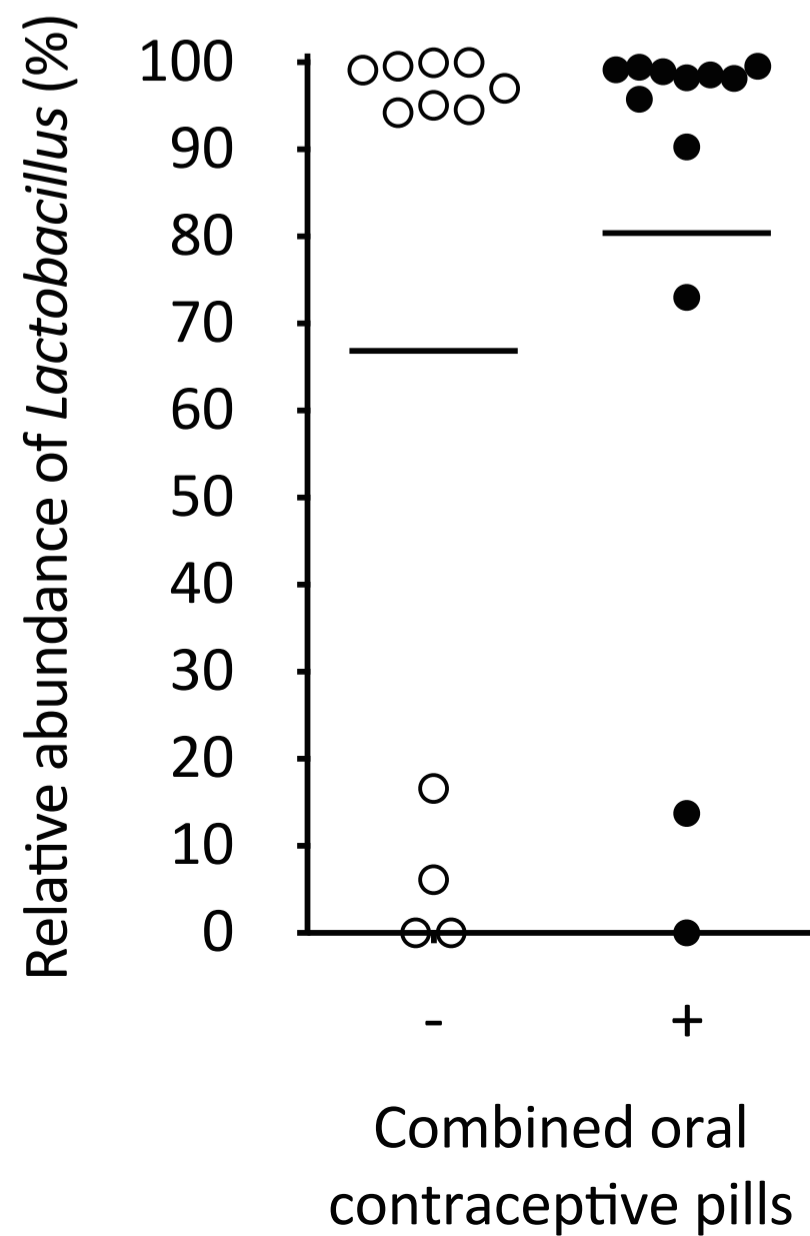**C**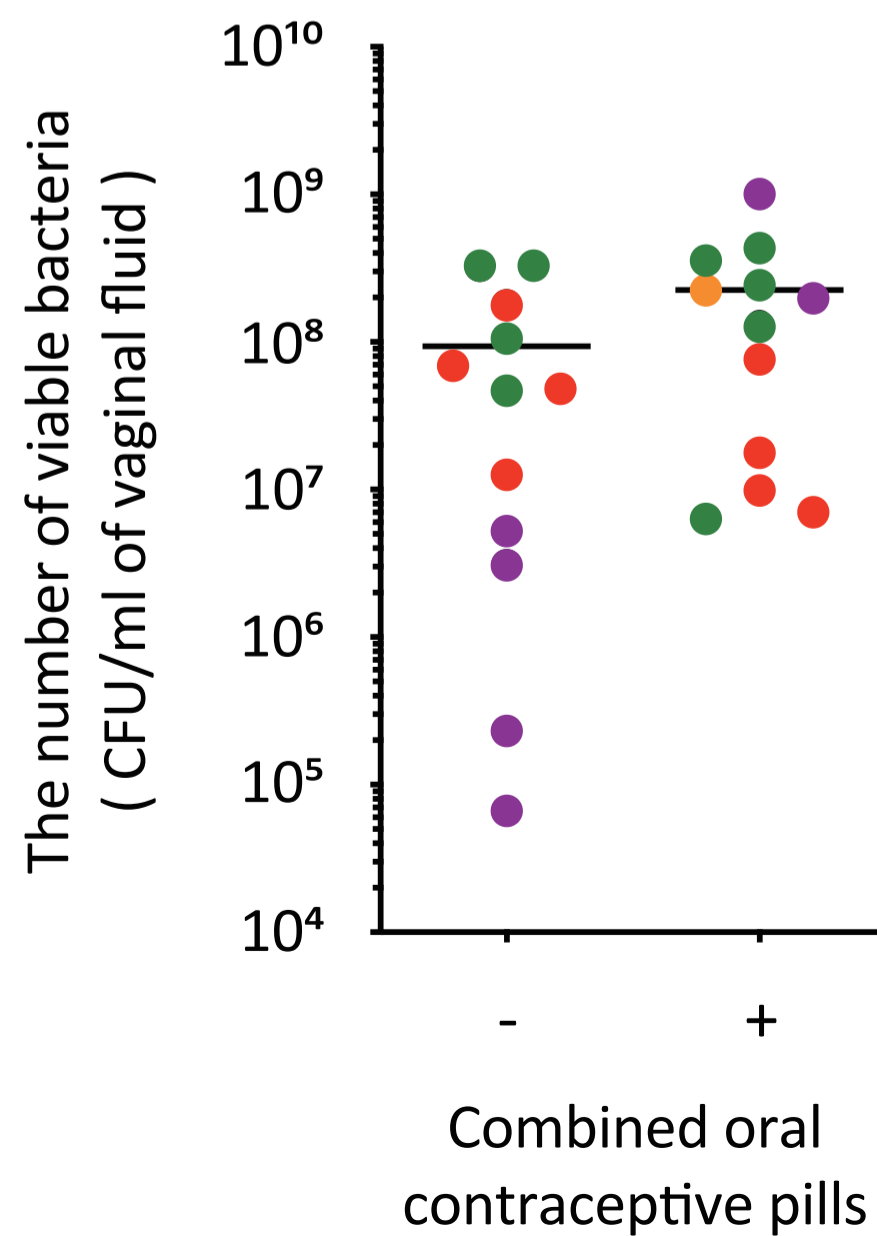**D**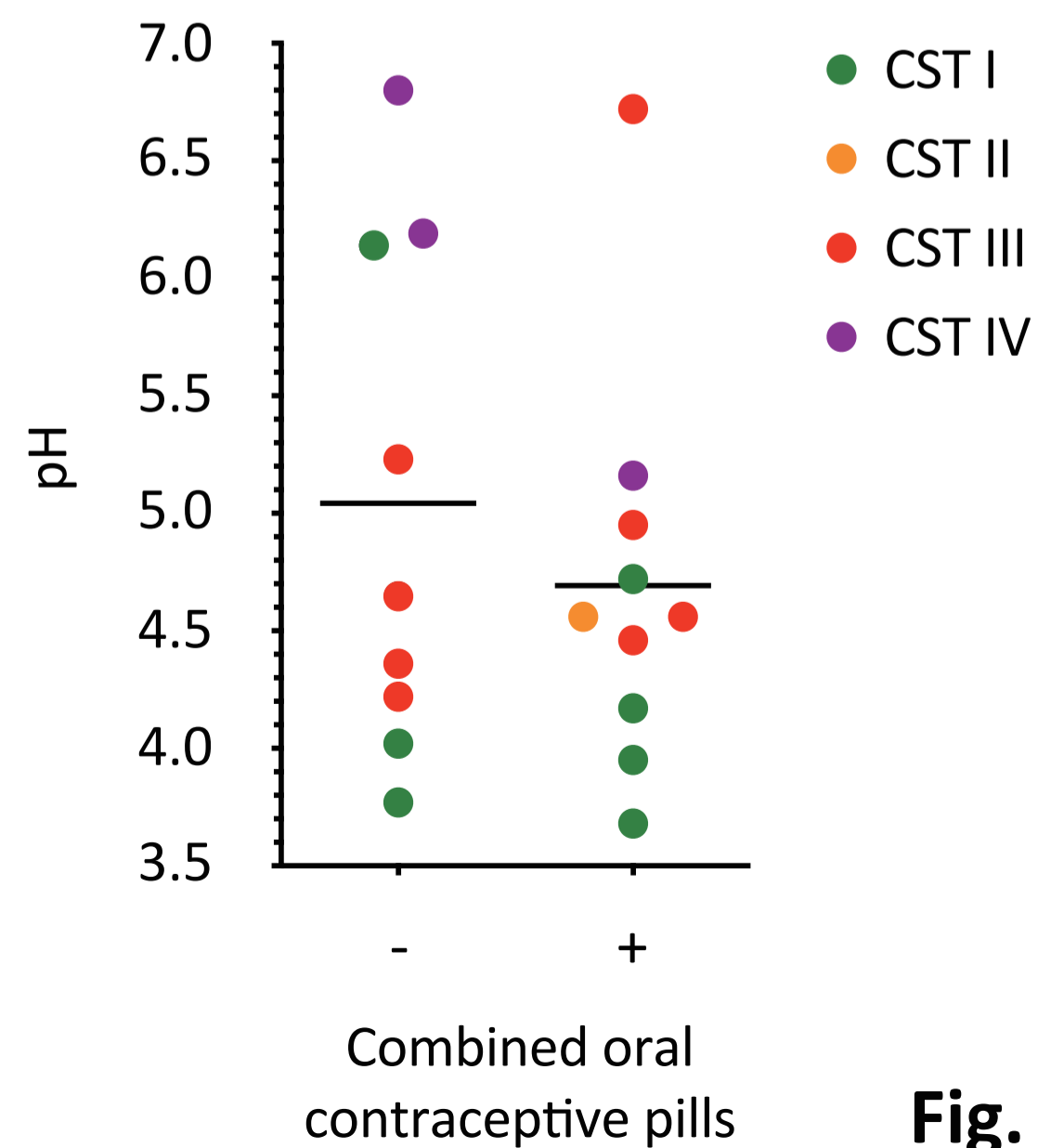**Fig. S1**

**A**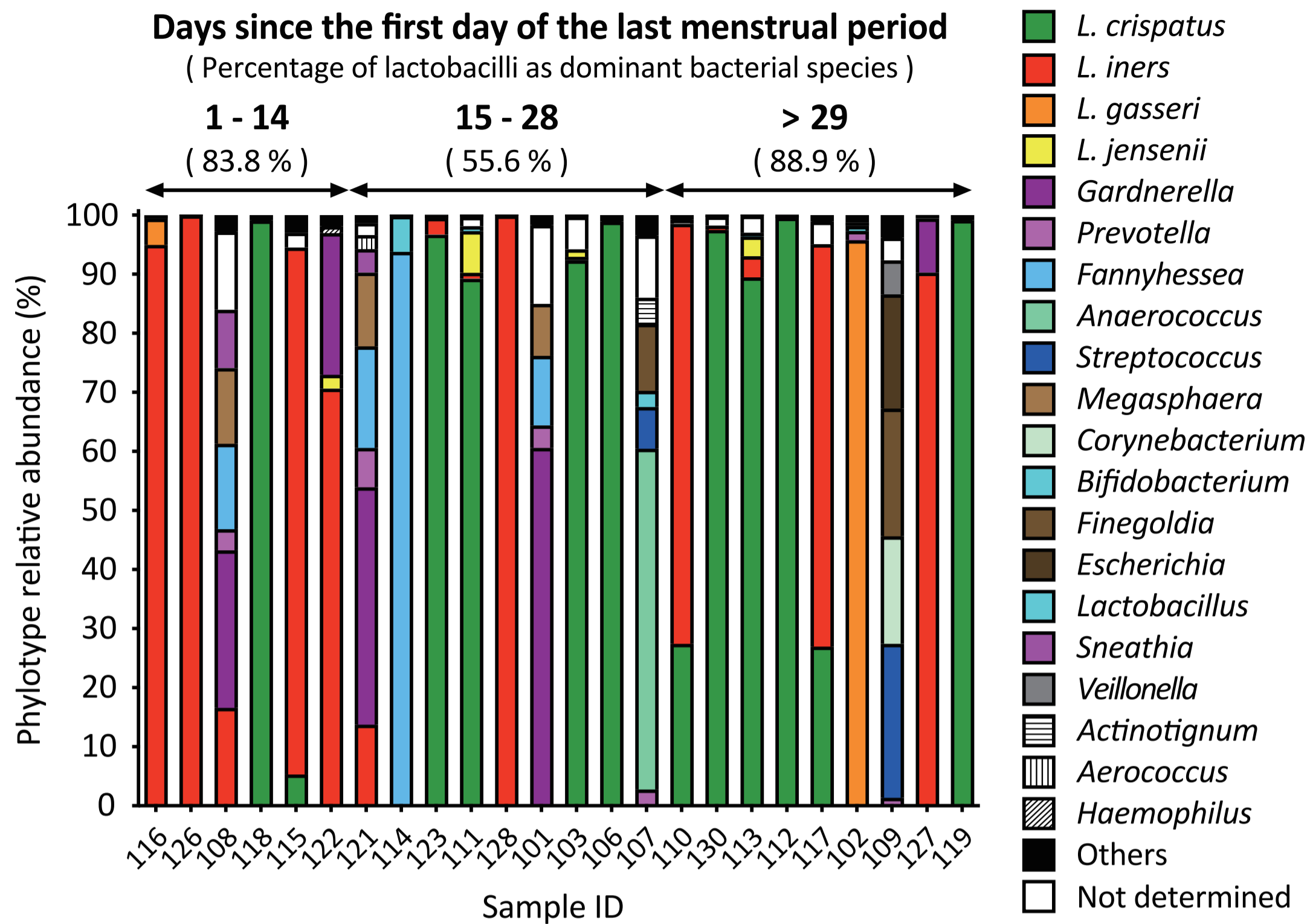**B**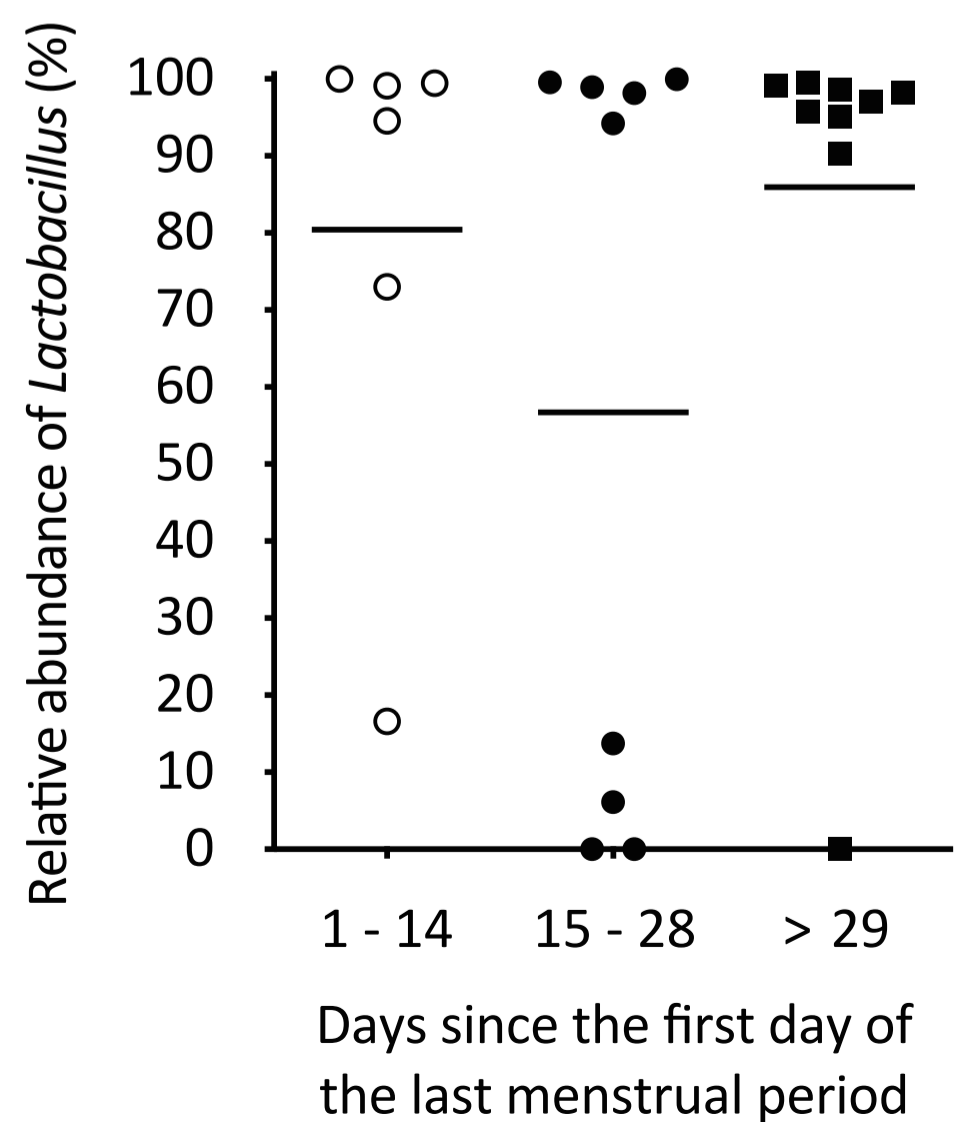**C**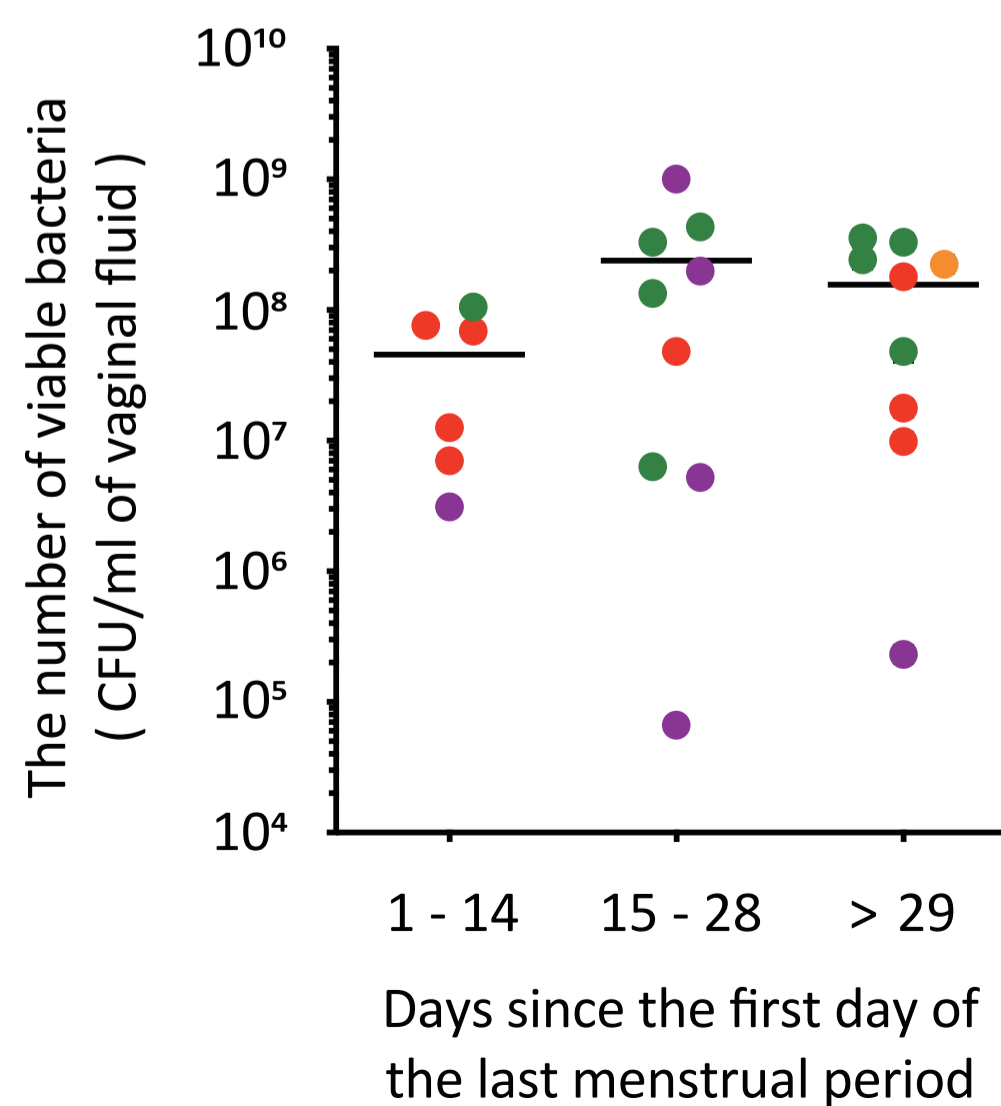**D**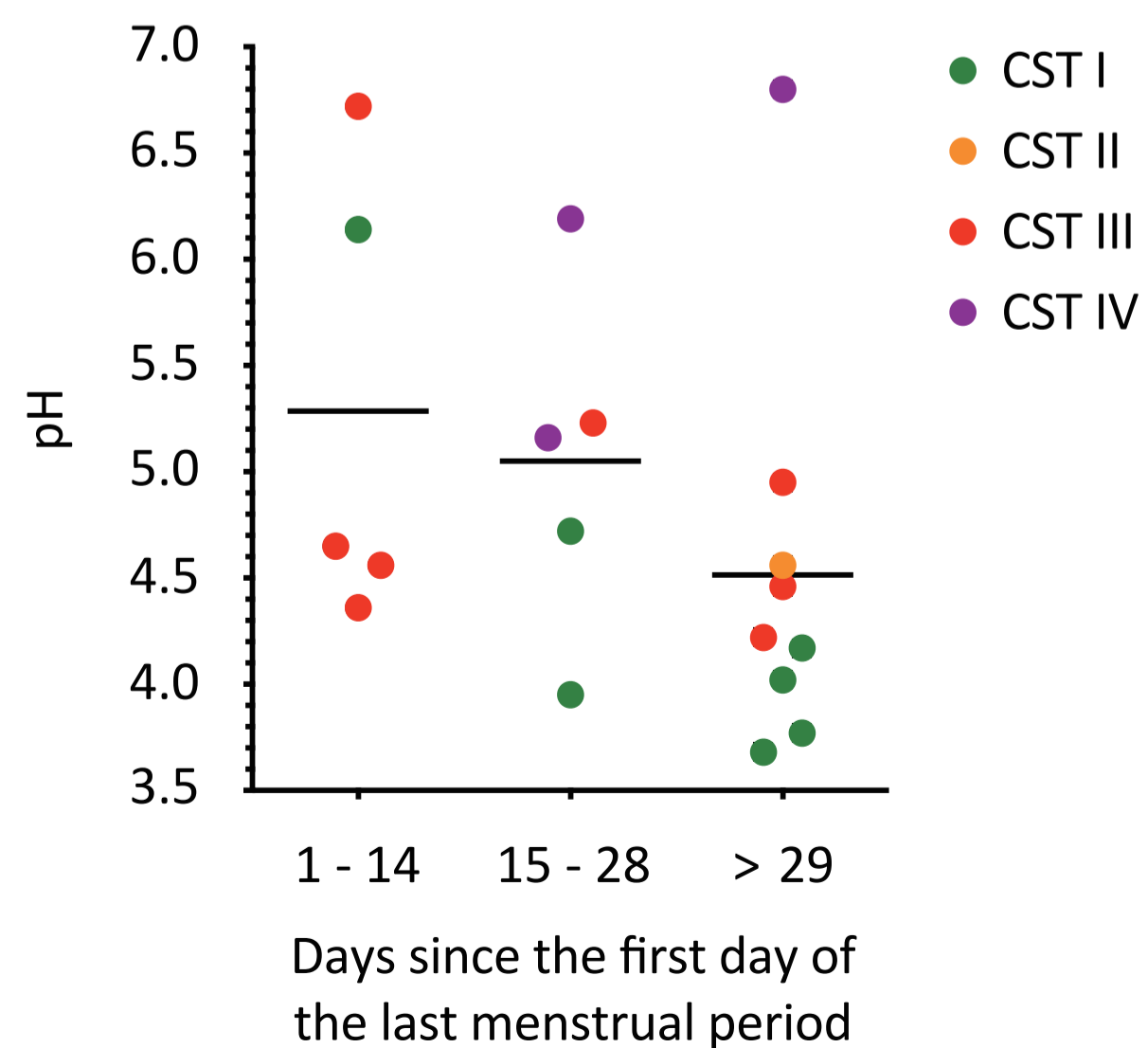**Fig. S2**

Supplement: Supplementary file 2 [file DataSheet2.pdf]
